# The Human Skin Microbiome in Selected Cutaneous Diseases

**DOI:** 10.3389/fcimb.2022.834135

**Published:** 2022-03-07

**Authors:** Silvia Carmona-Cruz, Luz Orozco-Covarrubias, Marimar Sáez-de-Ocariz

**Affiliations:** Department of Dermatology, Instituto Nacional de Pediatría, Mexico City, Mexico

**Keywords:** skin microbiome, atopic dermatitis, seborrheic dermatitis, alopecia areata, psoriasis, acne

## Abstract

The human skin harbors a wide variety of microbes that, together with their genetic information and host interactions, form the human skin microbiome. The role of the human microbiome in the development of various diseases has lately gained interest. According to several studies, changes in the cutaneous microbiota are involved in the pathophysiology of several dermatoses. A better delineation of the human microbiome and its interactions with the innate and adaptive immune systems could lead to a better understanding of these diseases, as well as the opportunity to achieve new therapeutic modalities. The present review centers on the most recent knowledge on skin microbiome and its participation in the pathogenesis of several skin disorders: atopic and seborrheic dermatitis, alopecia areata, psoriasis and acne.

## Introduction

The skin constitutes the external barrier between the human body and the environment. Considering the appendages, it has an estimated area of 25 m^2^ (Gallo, 2017). Regional variations in skin temperature, humidity, sebaceous gland density, and pH create different ecological niches where bacteria, fungi, viruses, archaea, and mites can thrive (Grice and Segre, 2011; Sanford and Gallo, 2013; Dréno et al., 2016; Gallo, 2017; Byrd et al., 2018; Lunjani et al., 2019).

**Figure 1:**
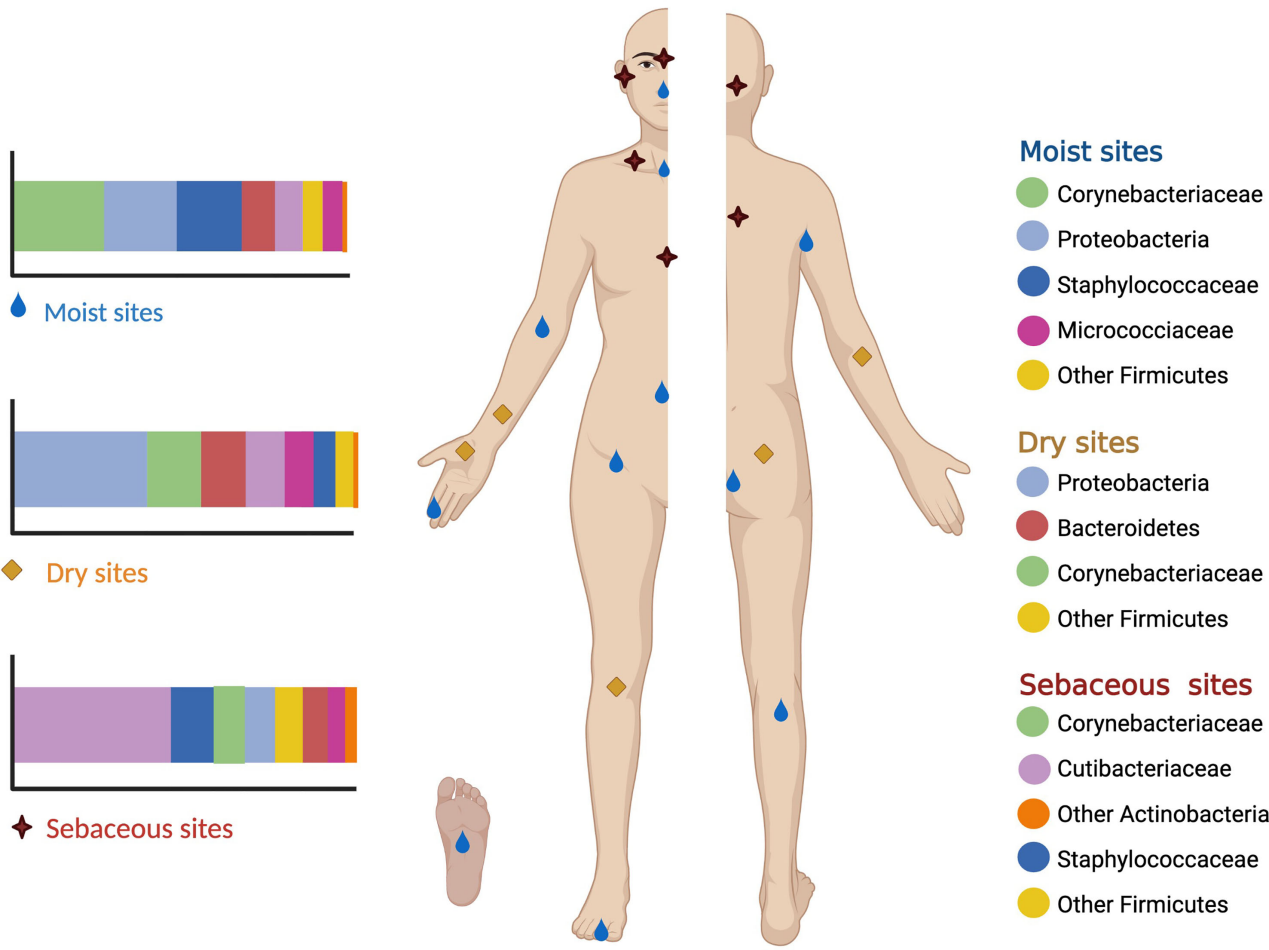
Moist sites include the axilla, antecubital fossa, navel, groin, popliteal fossa and soles. Oily sites include the forehead, alar creases, retroauricular creases and the back. Dry sites of the skin include the forearms, hands, buttocks and legs.

The human skin microbiome comprises the microorganisms together with their genetic elements and environmental interactions. Actinobacteria (36-51%), Firmicutes (24-34%), Proteobacteria (11-16%), and Bacteroidetes (6-9%) (Gallo, 2017; Byrd et al., 2018; McLoughlin et al., 2021) are the four major bacterial phyla found on the skin. In moist sites, the most abundant bacteria are *Staphylococcus* (Firmicutes) and *Corynebacterium* (Actinobacteria). Oily sites harbor the least diverse population where *Cutibacterium* (Actinobacteria) species are the most common isolates. Dry areas of the skin show the greatest diversity with varied colonization of the four phyla (Grice and Segre, 2011; Sanford and Gallo, 2013; Dréno et al., 2016; Byrd et al., 2018; Lunjani et al., 2019; McLoughlin et al., 2021; Rozas et al., 2021) (**Figure 1**).

Fungal species include *Malassezia* spp., *Cryptococcus* spp.*, Rhodotorula* spp.*, Aspergillus* spp., and *Epicoccum* spp. *Malassezia* spp. are the most common, comprising around 80% of the whole fungal flora (Grice and Segre, 2011; Sanford and Gallo, 2013; Dréno et al., 2016; Byrd et al., 2018; Lunjani et al., 2019; McLoughlin et al., 2021; Rozas et al., 2021). *Demodex* spp. are tiny mites living in the pilosebaceous follicles (Boxberger et al., 2021; Forton and De Maertelaer, 2021). Viruses have been the less investigated elements of the skin microbiota, cutaneous β and γ human papillomaviruses are commonly found on the skin surface but are scarce contrasted to the phages of the bacterial flora inhabiting the skin (Boxberger et al., 2021).

Microbiota colonization begins at birth and its composition is influenced by the route of delivery (Grice and Segre, 2011; Chu et al., 2017). Afterward, the composition is determined by several *intrinsic* (skin site, intra– and interpersonal variability, ethnicity, gender, and age) and *extrinsic *(lifestyle, hygiene routine, cosmetic use, antibiotics, geographical location, climate, and seasonality) factors (Giacomoni et al., 2009; Ursell et al., 2012; Sanford and Gallo, 2013; Pinto et al., 2021).

The skin microbiome has essential roles in the maintenance of skin homeostasis, the protection against invading pathogens, and the modulation of the immune system (Sanford and Gallo, 2013; Byrd et al., 2018; Boxberger et al., 2021).

Dysbiosis refers to the lack of balance among microbial communities within certain areas of the body that may lead to the onset or progression of diseases (McLoughlin et al., 2021). Several skin diseases such as atopic and seborrheic dermatitis, acne, alopecia areata, psoriasis and acne may result from dysbiosis (Sanford and Gallo, 2013; Dréno et al., 2016; Byrd et al., 2018).

## Atopic Dermatitis

Atopic dermatitis (AD) is a chronic inflammatory dermatosis that affects between 15–20% of children and 2–10% of adults (Lunjani et al., 2019). AD results from an intricate interaction between genetic susceptibility, barrier dysfunction, innate and adaptive immunity, and microbiome (Byrd et al., 2017).

*S. aureus* colonization is common in AD (Langan et al., 2020) with percentages varying from lesional (70%) to non–lesional (39%) sites (Hon et al., 2016; Totté et al., 2016; Byrd et al., 2017; Paller et al., 2019), *S. aureus* strains in AD patients differ from those isolated from asymptomatic carriers. However, some patients do not exhibit *S. aureus* overexpression, demonstrating the variability of AD (Hon et al., 2016; Fyhrquist et al., 2019).

Fyhrquist et al showed an increased abundance of *S. aureus* with depletion of *S. epidermidis* and *Corynebacterium* spp. among AD patients when compared to healthy controls (Fyhrquist et al., 2019). Further, genome–based assays demonstrated a change in the microbiome of AD patients before an outbreak, with loss of the diversity of cutaneous commensals and a predominance of *S. aureus* (Byrd et al., 2017; Paller et al., 2019; Woo and Sibley, 2020), the diversity returns to baseline once the disease is controlled (Kong et al., 2012; Fyhrquist et al., 2019; Langan et al., 2020). Whether *S. aureus* triggers AD or thrives because of the disease remains to be elucidated (Lunjani et al., 2019).

*S. epidermidis*, a commensal present on non–inflamed skin, appears to be *S. aureus* best antagonist (Woo and Sibley, 2020). *S. epidermidis* is thought to maintain skin microbiome balance by integrating innate immune pathways that control the function of effector T cells and exerting an antimicrobial function through the production of IL–1α by dendritic cells and keratinocytes, therefore limiting the ability of pathogens to establish infections (Paller et al., 2019). Further, the JK16 strain inhibits S. aureus biofilm formation (Hon et al., 2016). On clinical grounds, Byrd et al. showed that the less severe flares of AD had higher counts of *S. epidermidis* whereas the more severe flares were associated with *S. aureus* (Byrd et al., 2017).

*S. aureus* exploits AD–associated skin barrier defects with diminished antimicrobial peptides (β defensins, LL–Paulino, 2017, and dermcidin) and the low acid environment to achieve its colonization (Paller et al., 2019). *S. aureus–*derived toxins and proteases further damage the skin barrier and induce adaptive and innate immune responses (Sen et al., 2016; Nakagawa et al., 2017; Geoghegan et al., 2018; Lunjani et al., 2019) (**Table 1**).

**Table 1:** Staphyloccous aureus mechanisms of damage in atopic dermatitis.

*S. aureus* products	Mechanism of action
Alpha toxin	• Activation of the inflammasome through the secretion of IL1β • Interaction with ADAM 10➔ cadherin cleavage of the keratinocytes’ tight junctions (Woo and Sibley, 2020) • Formation of biofilms • Modulate the host´s response to viral infections (Hon et al., 2016)
Delta toxin	• Mast cell degranulation and differentiation to a Th2 phenotype (Langan et al., 2020; Woo and Sibley, 2020)
Staphylococcal lipoproteins	• Expression of thymic stromal lymphopoietin in keratinocytes, *via* TLR2/TLR6 receptors➔ Th2 phenotype (Langan et al., 2020)
Incorporation of unbranched fatty acids	• Evade the host´s immune response (greater fluency and the expression of virulence factors) • Resistance to oxidative stress by staphyloxanthin (Sen et al., 2016)
Phenol–soluble–modulin–α (PSM–α)	• Epidermal compartment PSMs stimulate IL–36α – driven Tγδ cell– mediated inflammation • In the dermal compartment they stimulate IL–1β – driven Th17 inflammation. • Stimulate cells and amplify inflammation (Paller et al., 2019) • Cytotoxic activity for keratinocytes (Nakagawa et al., 2017)

*Malassezia* spp. colonization increases with AD severity and has been detected in up to 90% of skin lesions. Its pathophysiological role may be due to the activation of pro–inflammatory cytokines and autoreactive cells that increase the expression of TLR2 and TLR4 through the secretion of immunogenic proteins (Lunjani et al., 2019; Langan et al., 2020).

Antimicrobial agents eradicate *S. aureus* however, they also affect other members of the skin microbiome, disrupting the homeostasis between species (Hon et al., 2016; Fyhrquist et al., 2019; Woo and Sibley, 2020) and generating bacterial resistance (Harkins et al., 2018).

Biotherapy uses the “protective” effect of commensal agents against pathogens to improve dysbiosis in patients with AD (Woo and Sibley, 2020). *Staphylococcus hominis*, *S. lugdunensis and S. epidermidis* produce several molecules capable of synergizing the innate antimicrobial response against *S. aureus* (Byrd et al., 2017).

The beneficial effect and exact mechanism of action of biotherapy are still under investigation. Murine studies and some clinical trials, in which commensal bacteria were applied topically, showed improvement in both AD models and disease severity scales (Nakatsuji et al., 2017; Nakatsuji et al., 2018; Myles et al., 2018). A randomized 1–week trial of topical *S. hominis* A9 (ShA9) – isolated from healthy human skin – or vehicle on the forearm skin of 54 adults with *S. aureus* positive AD, met the primary endpoint of safety and resulted in a reduction of *S. aureus* colony unit formation in both lesional and non–lesional skin during treatment and up to 96 hours after discontinuation (Nakatsuji et al., 2021). Further, a double–blind, vehicle–controlled randomized clinical trial evaluated in 11 adult patients with moderate/severe AD if the autologous topical application of antimicrobial–producing coagulase negative *Staphylococcus* (CoNS) obtained from non–lesional skin of AD patients could inhibit S. aureus and improve clinical outcomes. S. aureus colonization was reduced (by 99.2%) and EASI scores improved in patients thar received active treatment compared with vehicle (Nakatsuji et al., 2021).

Other therapies directed to the dysbiosis include narrow–band UVB phototherapy – which decreases the number of *S. aureus* and its production of superantigens – (Woo and Sibley, 2020) and the development of vaccines against *S. aureus* (Clowry et al., 2019).

Regarding the intestinal microbiome, a low diversity within the first weeks of life confers a higher risk of developing AD (Lee et al., 2018). Comparative studies of intestinal microbiome between patients with AD and healthy controls have revealed that *Faeclibacerium prausnitzii* and *Ruminocococus gnavus* are increased in patients with AD, whereas *Bacteroides fragilis* and *Streptococcus salivaris*, are less abundant (Zheng et al., 2016). Therefore, those interventions that increase microbial diversity using prebiotics, probiotics, or symbiotics could prevent AD from developing in high–risk children.

## Seborrheic Dermatitis

Seborrheic dermatitis (SD) is a common chronic inflammatory skin disease in both pediatric and adult ages, ranging from slight scalp scaling to severe erythematous plaques of the scalp, face, and trunk (Chen et al., 2021).

The direct correlation of the number of yeasts with the severity of the disease and the symptomatic improvement of the affected skin after the use of antifungals support *Malassezia’s* relevant role in the pathogenesis of SD (Tanaka et al., 2016; Chen et al., 2021), however, some studies disagree with the findings (Yu et al., 2020a). Thus, *Malassezia’s* overgrowth might be important only in predisposed individuals due to differences in sebaceous gland function, immune function, and lipid composition (Pedrosa et al., 2014; Prohic et al., 2015; Adalsteinsson et al., 2020).

Malassezia preferentially colonizes seborrheic areas of the skin, where it uses saturated fatty acids and disregards unsaturated fatty acids (oleic acid), that disrupt the barrier function and condition an inflammatory response in the skin (Tanaka et al., 2016; Xu et al., 2016; An et al., 2017; Pedrosa et al., 2018; Adalsteinsson et al., 2020; Chen et al., 2021).

The interaction of *Malassezia* with epidermal cells, in susceptible individuals, stimulates antigen–presenting cells through pattern recognition receptors – TLR–(Byrd et al., 2018), NOD–like, and C–type lectin receptors (Pedrosa et al., 2018; Adalsteinsson et al., 2020; Chen et al., 2021) –, that activate several cell–signaling pathways such as MPAK, NF–κB, and NFAT leading to inflammation and secretion of pro–inflammatory cytokines and mediators. The inflammation further increases barrier dysfunction and dysbiosis (Ro and Dawson, 2005; Naldi and Rebora, 2009; Schwartz et al., 2013).

Sebaceous areas are also colonized by several bacteria whose role has been recently studied (Wikramanayake et al., 2019). Paulino et al. compared the bacterial and fungal microbiota of lesional and non–lesional skin in healthy controls and SD patients, finding greater diversity and variation in both bacteria and fungi in SD patients (Paulino, 2017). Besides, Tanaka et al. studied the bacterial microbiota in lesional and non–lesional sites of 24 patients with SD, finding a predominance of *Acinetobacter*, *Staphylococcus*, and *Streptococcus* in lesional skin, whereas *Cutibacterium* predominated in non–lesional skin, and colonization by *Corynebacterium* was similar in both (Tanaka et al., 2016). Higher colonization rates of *S. epidermidis* have been found in facial seborrheic dermatitis in Chinese patients (An et al., 2017; Adalsteinsson et al., 2020), and in HIV positive and negative patients with SD (Pedrosa et al., 2018). One more recent report has mentioned that *S. aureus* is the most common bacterial member of the skin flora in patients with SD (Tamer et al., 2018; Adalsteinsson et al., 2020).

Several studies have shown an increase in the *Malassezia restricta/Malassezia globosa* ratio and a reduction in the *Cutibaterium/Staphylococcus* ratio in SD. *Staphyloccus* is linked with epidermal barrier damage, including increased transepidermal water losses and pH, while *Cutibacterium* increases water content. *Malassezia* has also been associated with increased pruritus and disease severity (Xu et al., 2016; Park et al., 2017; Tao et al., 2021).

Some studies have focused on the use of probiotics to treat SD (Yu et al., 2020a). Guéniche et al. showed a decrease in erythema, pruritus, and scaling with the topical application of *Vitreoscilla filiformis* in 60 patients (Guéniche et al., 2008). Besides, the oral administration of *Lactobacillus paracasei* improved erythema, seborrhea, and dandruff in patients with SD (Reygagne et al., 2017). The therapeutic effect may be related to a rise in the activity of regulatory T cells, and the secretion of IL–10 and transforming growth factor–beta by dendritic cells (Yu et al., 2020a).

## Alopecia Areata

Alopecia areata (AA) is a nonscarring type of hair loss with an incidence of 2% and a greater prevalence among pediatric populations. Hair loss can range from well–defined patches to diffuse or total hair loss, which can affect all hair–bearing sites (Strazzulla et al., 2018).

The pathogenesis of AA remains incompletely understood. It is believed that autoimmune–mediated HF destruction, the upregulation of inflammatory pathways, and loss of immune privilege in the hair follicle (HF) result in AA (Strazzulla et al., 2018; Anzai et al., 2019). Genetic predisposition, environmental factors, and recently the skin and gut microbiome have been related to autoimmunity in AA (Rebello et al., 2017; Migacz–Gruszka et al., 2019; Simakou et al., 2019; Juhasz et al., 2020).

The HF microbiota is located near the bulge (stem cell niche) and the bulb (cellular division site to build a new hear) considered immune–privileged sites. Shifts in the HF microbiome can be related to loss of homeostasis, modulation of immune reactions and the intense peribulbar inflammation in AA (Colucci and Moretti, 2021).

Symbiosis of *Corynebateriaceae*, *Propionibacteriaceae*, Staphylococcaceae, and *Malassezia* is related to a healthy scalp, while dysbiosis can cause pathological conditions. Pinto et al. found microbial shifts in individuals with AA exhibiting over–colonization with *C. acnes* along with a reduced *S. epidermidis* abundance, however, it has not been determined if these differences are cause or consequence of the disease (Pinto et al., 2019). The role of CMV in triggering AA was suggested after finding DNA sequences in biopsies of AA, but subsequent studies did not confirm this fact (Offidani et al., 2000). Rudnicka et al. postulated a possible relation between scalp colonization by *Alternaria* spp. and AA after it was cultured from epidermal scrapings in patients (Rudnicka and Lukomska, 2012).

Along with skin, gut dysbiosis has been recently linked with AA (Polak–Witka et al., 2020; De Pessemier et al., 2021). Genes that are related to AA can affect gut colonization with microorganisms that induce a Th1 response with increased IFNγ production. There are two cases of AA with long–term hair regrowth after fecal microbiota transplants that support a role of the intestinal microbiome (Rebello et al., 2017) in the pathophysiology of AA. Nonetheless, certain gut microbiota differences identified in patients with AA were not significant (De Pessemier et al., 2021).

AA is associated with other autoimmune disorders. Gut dysbiosis can act as a common pathway in patients with both inflammatory bowel disease and AA. A variety of factors involved in hair growth and/or maintaining immunological homeostasis are affected in gut dysbiosis: bacterial production of biotin, short–chain fatty acids produced by gut microbiota, vitamin D deficiency, among others (Moreno–Arrones et al., 2020). Restoration of gut microbiota balance might contribute to hair regrowth in patients with alopecia areata by enhancing the absorption and synthesis of nutrients and host–related factors such as immunomodulation (Xie et al., 2019).

An innovative treatment option for AA is the therapeutic manipulation of the microbiome. This manipulation may be achieved by fecal microbiota transplant (Rebello et al., 2017; Xie et al., 2019) or the use of microbial metabolites such as postbiotics (Rinaldi et al., 2020).

## Psoriasis

Psoriasis vulgaris (PV) is a chronic inflammatory disorder with a worldwide prevalence of 2% (Hugh and Weinberg, 2018). PV results from a complex interaction between genetic predisposition and environmental factors that incite immune dysregulation and trigger a rapid proliferation of keratinocytes and infiltration of immune cells with the formation of erythematous and scaly plaques (Hugh and Weinberg, 2018; Thio, 2018; Visser et al., 2019; Navarro–López et al., 2019; McLoughlin et al., 2021; Chen et al., 2021).

The breakdown of immune tolerance to cutaneous microorganisms has been implicated in the pathogenesis of PV (Lewis et al., 2019). Bacteria found on PV plaques include Firmicutes, Actinobacteria, and Proteobacteria (Alekseyenko et al., 2013). Other authors have shown a decrease in Actinobacteria and Bacteroides and an increase in *Coprobacillus, Ruminococcus*, and *Streptococcus* (Thio, 2018; Visser et al., 2019).

Several studies have shown increased numbers of *Staphylococcus* in both non–lesional (*S. sciuri* and *S. aureus*) (Chang et al., 2018) and lesional skin (*S. aureus* and *S*. ptettenkoferi) (Tett et al., 2017; Chang et al., 2018) when compared to the skin of healthy individuals. Whereas *S. epidermidis*, *C. acnes* and *C. granulosum* are more numerous in healthy than in psoriatic skin (Gao et al., 2008; Alekseyenko et al., 2013; Tett et al., 2017; Chang et al., 2018).

*S*. *aureus* colonizes psoriatic lesions in 60% of the patients and up to 60% (Lewis et al., 2019) secrete enterotoxins and toxic shock syndrome toxin–1. Colonization by *S. aureus* is thought to trigger an inflammatory Th17 response responsible for the perpetuation of keratinocyte proliferation (Elfatoiki et al., 2016; Chang et al., 2018).

*S. pyogenes* is also frequently identified as trigger for both the development and the exacerbations of PV (Hugh and Weinberg, 2018; Lewis et al., 2019). Pharyngeal infection by *S. pyogenes* induces superantigen activation of T cells. Th1 cells recognize group A *Streptoccocus* wall antigens and produce IFNγ, which is elevated in up to 70% of patients with guttate and chronic plaque psoriasis lesions. Additionally, superantigens located in the dermal layers bind directly to HLA–DR receptors on dendritic cells, macrophages, and keratinocytes, perpetuating inflammation (Lewis et al., 2019).

The association of *Malassezia* with PV is unclear (Chen et al., 2002; Lewis et al., 2019; Mazur et al., 2021). Its alleged involvement relies on *Malassezia’s* ability to invade keratinocytes which in turn increase the expression of TGFβ, integrin chains. and heat shock protein 70 that induce hyperproliferation. Besides, by the secretion of chemotactic factors, *Malassezia* attracts neutrophils to PV lesions (Lewis et al., 2019; Mazur et al., 2021).

*Candida albicans* has been linked to skin lesion persistence and worsening, notably in inverse psoriasis (Waldman et al., 2001; Lewis et al., 2019). The mechanism remains unknown, however, it may be mediated by superantigens (Fry and Baker, 2007).

Regarding viruses, patients infected by human immunodeficiency virus or human papillomavirus, have more severe features of psoriasis related to the secretion of substance P that stimulates the proliferation of keratinocytes (Chen et al., 2002; Lewis et al., 2019; Chen et al., 2021).

Several studies suggest that the intestine is the possible origin of the dysbiosis observed in psoriasis (Fry and Baker, 2007). The most frequently reported findings are the reduction of *Bacteroides*, and *Akkermansia* spp. and the increase of Firmicutes and Actinobacteria (Navarro–López et al., 2019; Chen et al., 2021).

The altered intestine leads to bacterial translocation, triggering inflammation and an aberrant microbiome, which produces a perpetuation of the inflammatory response (Ramírez–Boscá et al., 2015). Inflammatory bowel disease and psoriasis have an association (Thio, 2018; Visser et al., 2019), the presence of Crohn’s disease increases up to 5 times the risk of developing psoriasis (Lewis et al., 2019). In these patients, *Faecalibacterium praunitzii*is is reduced, limiting its anti–inflammatory effect through the inhibition of the NFκB pathway (Visser et al., 2019).

The use of antibiotics to attack dysbiosis has shown improvement of psoriasis (Saxena and Dogra, 2005; Yu et al., 2020b) but with the cost of eradicating beneficial communities both in the intestine and on the skin (Visser et al., 2019).

Probiotics have an immunomodulatory effect by increasing the production of immunoglobulins or activating lymphocytes and mononuclear cells (Yu et al., 2020b). Navarro–López observed among 90 patients with psoriasis that the group of 45 patients that received mixed probiotics (*Bifidobacterium longum*, *B. lactis*, and *Lactobacillus rhammosus*) had a decrease in the severity of psoriasis, a better response rate to treatment, less need for steroids, and a lower risk of relapse (Navarro–López et al., 2019).

The use of prebiotics and symbiotics may have a beneficial effect as adjunctive treatments in psoriasis by modifying the inflammatory responses of CD4 + and CD8 + T lymphocytes (Chen et al., 2002). The role of fecal microbiota transplantation is under investigation to modify the intestinal microbiota and serve as therapy in psoriasis and its comorbidities (Mazur et al., 2021).

## Acne

Acne is a chronic inflammatory disorder of the pilosebaceous follicle affecting more than 85% of adolescents and young adults. Its pathogenesis includes increased sebum production, follicular hyperkeratinization, skin microbiome, and *Cutibacterium acnes*, and inflammation (Knutsen–Larson et al., 2012).

Acne has been largely associated with *C. acnes *proliferation. However, several authors have found that the abundance and bacterial load of C. acnes do not differ significantly between acne and healthy patients (Miura et al., 2010; Fitz–Gibbon et al., 2013; Dessinioti and Katsambas, 2017; Omer et al., 2017; Li et al., 2019). Thus, it appears that the loss of the diversity between the six phylogenetic groups (IA_1,_ IA_2_, IB, IC, II, and III) with predominance of IA_1_ and to a lesser degree IA_2_, rather than *C. acnes* proliferation plays a role in the triggering of acne (Pécastaings et al., 2018; Dagnelie et al., 2018; Corvec et al., 2019; McLaughlin et al., 2019; Dagnelie et al., 2019).

*C. acnes *triggers the release of proinflammatory cytokines after binding to TLR–2 that activate NLRP3 inflammasomes and caspase1–, driving the secretion of IL–1β, T cell differentiation and lymphocyte and neutrophil recruitment to acne lesions. TLR–2 activation also stimulates IL–1α production with a pivotal role in comedogenesis through the stimulation of keratinocyte proliferation (Dreno, 2017). *C. acnes* can also form biofilms that increase virulence and resistance to antimicrobial treatments (Dreno, 2017; Hazarika, 2021).

*S.epidermidis* inhibits *C. acnes* proliferation by favoring the fermentation of glycerol and releasing succinic acid (Dreno et al., 2017; Claudel et al., 2019). It also reduces C. *acnes*–induced skin inflammation by the production of lipoteichoic acid which inhibits TLR2 production, and IL–6 and TNFα by keratinocytes (Skabytska and Biedermann, 2016). On the other hand, *C. acnes *inhibits S. *epidermidis* proliferation by keeping the acidic environment of the pilosebaceous follicle, hydrolyzing sebum triglycerides, and secreting propionic acid (Dréno et al., 2020). The loss of balance between them induces the activation of inflammation–related markers (IL1ra, IL–Grice and Segre, 2011, IL–Rozas et al., 2021, G–CSF) and other molecules (C5/C5a, soluble CD14 MIP–3beta, Serpin E, VCAM–Gallo, 2017, and β−defensin 2) (Dagnelie et al., 2021).

*Malassezia* can also play a role in refractory acne. Its lipase, 100 times more active than that of *C. acnes*, attracts neutrophils and stimulates the release of pro–inflammatory cytokines from monocytes and keratinocytes. Its exact involvement in the pathogenesis of acne, however, has yet to be determined (Lee et al., 2019).

Tetracyclines are frequently the first choice of treatment for moderate/severe acne since they suppress *C. acnes *growth and control of inflammation (Dréno et al., 2020). However, the antimicrobial activity also modifies cutaneous microbiota that may be beneficial (Lam et al., 2021).

Currently, the development of a vaccine against CAMP virulence factor to induce immunity has resulted – in *ex vivo *models – in decreased growth of *C. acnes* and proinflammatory toxins (Wang et al., 2018).

Intermittent pulsed light improves the severity of acne, regulates the balance between *S. epidermidis* and C. acnes and inhibits sebum secretion (Liu et al., 2021). Recently, Yang et al. demonstrated, on 5 patients with moderate to severe acne, that photodynamic therapy increased the diversity of skin microbiome in acne and shifted follicular microbiome towards epidermal microbiome, exerting its beneficial effect partly by inhibiting *C. acnes* and modifying the composition and potential function of skin microbiome in acne (Yang et al., 2021).

## Discussion

Molecular approaches to define microbial diversity have modified our understanding of the skin microbiome and raised several questions regarding the host–microbe interaction and its relevance to skin disease. Current knowledge has shown that bacterial, fungal, and viral species are under or overexpressed in several dermatoses when compared with healthy skin. To date, the pathogenic role of several species has been suggested in diverse skin diseases, such as *C.acnes* in acne or *S. aureus* in AD. However, the paradigm of the microbiome related to some skin diseases has shifted from the proliferation of one or more microorganisms to the loss of diversity among several microorganisms in producing disease.

Despite the progress in identifying the variations of the skin microbiome, it is yet unclear if shifts in the microbiome play a causative role in the skin diseases here discussed or merely represent a consequence of the inflammatory microenvironment. Thus, more research is needed to fully understand how microbiome variations relate to the genetic and environmental factors that also contribute to the disease. Further analysis of microorganism interaction with the immune system and among each other, the relationship between the skin microbiome and other niches (such as the gut), and the modification on the growth conditions of microorganisms according with the host’s own intrinsic and extrinsic factors is still needed.

As we gain a better knowledge of the skin microbiota, several unanswered questions will guide future research efforts directed towards understanding the intricate interactions controlling the host–microorganism relation and the possibility of the host and the microorganism developing together.

Finally, as antimicrobial treatments directed against the pathogens associated with skin diseases also eradicate beneficial flora, investigation efforts have shifted to the maintenance of skin microbiota homeostasis through the utilization of probiotics, prebiotics, symbiotics, and fecal transplantation. In the close future, their real therapeutic effectiveness will certainly be established.

## Author Contributions

SC–C made substantial contributions on the acquisition of data for the work, revised it critically for important intellectual content, provided approval for publication of the content and agrees to be accountable for all aspects of the work in ensuring that questions related to the accuracy or integrity of any part of the work are appropriately investigated and resolved. LO–C made substantial contributions to the design of the work, revised it critically for important intellectual content, provided approval for publication of the content, and agreed to be accountable for all aspects of the work in ensuring that questions related to the accuracy or integrity of any part of the work are appropriately investigated and resolved. MS–d–O made substantial contributions to the conception of the work, drafted the work, provided approval for publication of the content, and agreed to be accountable for all aspects of the work in ensuring that questions related to the accuracy or integrity of any part of the work are appropriately investigated and resolved.

## Funding

This work was supported by the E022 Program from the Instituto Nacional de Pediatría.

## Conflict of Interest

The authors declare that the research was conducted in the absence of any commercial or financial relationship that could be construed as a potential conflict.

## Publisher’s Note

All claims expressed in this article are solely those of the authors and do not necessarily represent those of their affiliated organizations, or those of the publisher, the editors and the reviewers. Any product that may be evaluated in this article, or claim that may be made by its manufacturer, is not guaranteed or endorsed by the publisher.
